# Bowen’s Disease Along With Intradermal Merkel Cell Carcinoma Occurring on the Dorsum of the Hand

**DOI:** 10.7759/cureus.69114

**Published:** 2024-09-10

**Authors:** Chihiro Ishihara, Tadashi Nomura, Takaaki Nakanishi, Yoriko Tsuji, Hiroto Terashi

**Affiliations:** 1 Plastic Surgery, Kobe University Graduate School of Medicine, Kobe, JPN; 2 Diagnostic Pathology, Kobe University Graduate School of Medicine, Kobe, JPN

**Keywords:** bowen’s disease (bd), intradermal mcc, merkel cell carcinoma (mcc), squamous cell carcinoma (scc), subcutaneous mcc

## Abstract

Bowen’s disease (BD), also known as squamous cell carcinoma (SCC) in situ, is a precancerous skin condition that can potentially progress to invasive tumors. Merkel cell carcinoma (MCC) is a rare and aggressive neuroendocrine tumor typically found in sun-exposed areas of elderly Caucasians. The coexistence of BD and MCC is extremely rare, particularly when MCC is located in subcutaneous tissue rather than its typical epidermal or dermal layers. This case report presents an unusual instance of BD coexisting with subcutaneous MCC on the dorsum of the hand in an elderly Japanese male. An 87-year-old Japanese male with over 30 years of sun exposure presented with a progressively enlarging red tumor on the dorsum of his left hand. A biopsy confirmed BD, and the tumor was excised with a 5 mm margin followed by skin grafting. Histopathological examination revealed subcutaneous MCC along with BD, with MCC cells forming small nests in the dermal papillary layer. Immunohistochemistry showed positive staining for synaptophysin and CK20 in a perinuclear dot pattern, confirming the MCC diagnosis. Given the patient’s advanced age and the absence of positive surgical margins, a watch-and-wait approach was adopted. The patient has been under close outpatient monitoring, and no recurrence has been observed after six months. This case highlights the rarity of subcutaneous MCC coexisting with BD, with only a few reported cases of such coexistence. The unusual subcutaneous presentation and the presence of multiple micro-nodules instead of large atypical cell clusters suggest an early-stage MCC beneath BD. The pathogenesis of this coexistence remains unclear but raises important questions regarding the relationship between sun exposure and viral factors like Merkel cell polyomavirus (MCPyV), which was not tested in this case. The findings underscore the need for comprehensive diagnostic evaluation when encountering complex or atypical skin lesions. This report emphasizes the rarity of subcutaneous MCC coexisting with BD and underscores the importance of comprehensive diagnostic assessment in unusual cases. Further research is warranted to better understand the underlying mechanisms and to guide optimal management strategies for such rare and challenging presentations.

## Introduction

Bowen’s disease (BD), also recognized as squamous cell carcinoma (SCC) in situ, represents a gradually advancing precancerous skin ailment. Approximately 3%-5% of BD cases may advance to invasive tumors. The trunk and limbs were the most frequently affected areas for BD. Clinically, patients with BD often have concurrent other conditions such as cutaneous pseudo lymphoma and breast cancer [1].

Merkel cell carcinomas (MCCs) are aggressive tumors originating from neuroendocrine cells, usually presenting as solitary, dome-shaped red nodules or hardened plaques. Common sites for MCCs include the skin of the head and neck, which are sun-exposed areas in elderly Caucasian patients [2]. MCCs itself is a rare condition, and cases where BD and MCCs coexist are uncommon. Furthermore, the sites of onset for BD and MCC are also different. Therefore, case reports of Bowen's disease concurrent with Merkel cell carcinoma are exceedingly rare. Notably, in pathological findings, while BD and MCC predominantly originate within the epidermis, we encountered a case where MCC was found in the subcutaneous tissue. We report a case of BD with MCC in the subcutaneous tissue occurring on the dorsum of the hand in an elderly Japanese patient.

## Case presentation

**Figure 1:**
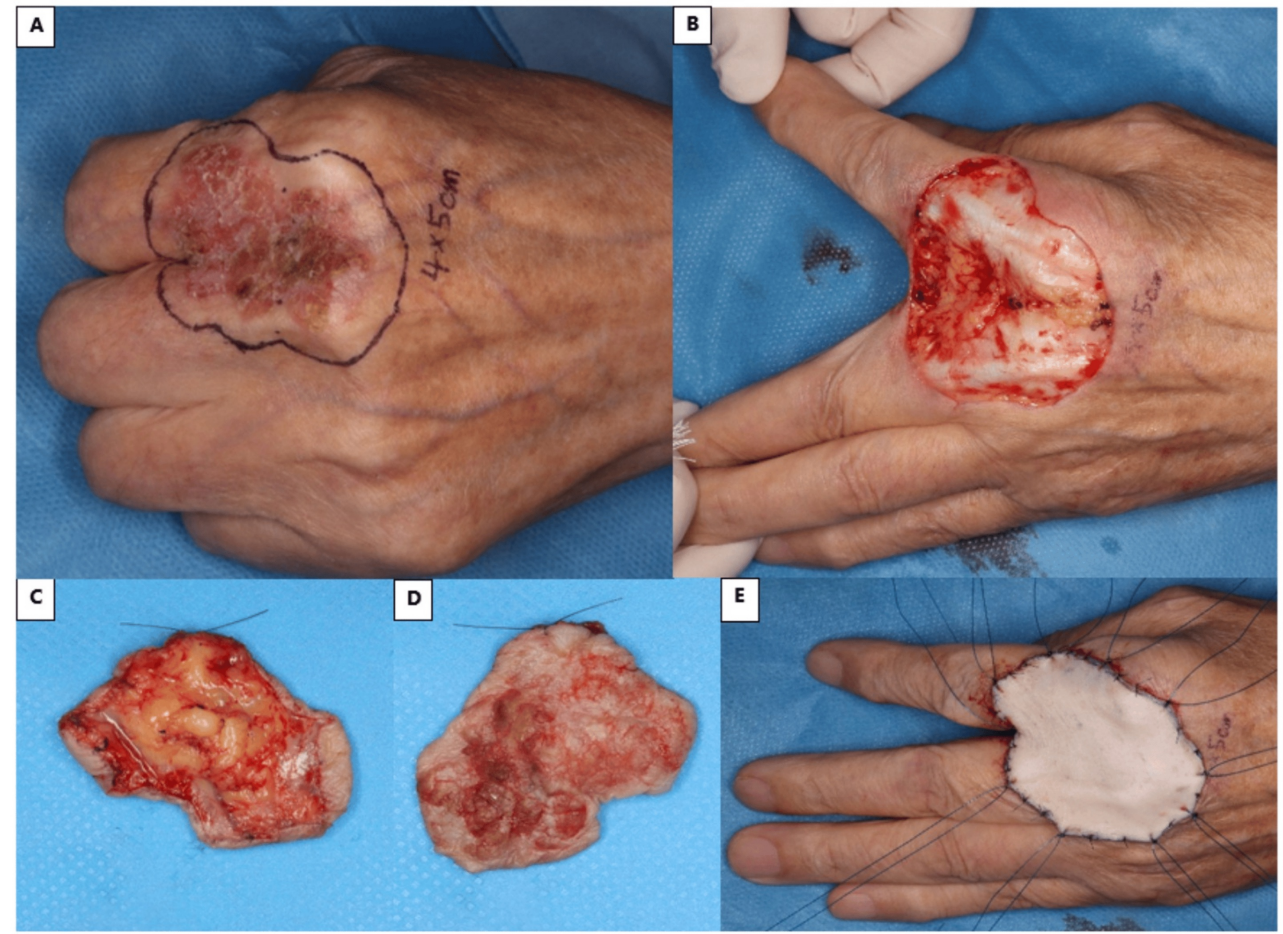
Surgical images A) There is a scaly and red tumor on the dorsum of the hand, and the design shows the 5 mm surgical margin. B) After the tumor resection, the extensor tendons were preserved. C) The adipose layer was exposed at the deep side of the tumor. D) Surface of the tumor. E) A skin graft harvested from the right inguinal region and fixed with tie-over sutures.

An 87-year-old Japanese male presented with a scaly and red tumor with well-defined borders on the dorsum of his left hand, which had been progressively growing in aggressiveness over the past year (Figure 1A). He had worked in the construction field, with over 30 years of exposure to sunlight and no history of arsenic exposure. He was prescribed steroid ointments by another physician. However, the symptoms did not improve and the size of the tumor was increasing and deteriorating. A partial excision biopsy was conducted by the physician, resulting in a diagnosis of Bowen’s disease. First, after excising the tumor and confirming negative margins, we considered performing a secondary skin graft; however, due to advanced age, the decision was made to pursue primary surgical intervention and subsequently perform skin grafting following tumor excision.

**Figure 2:**
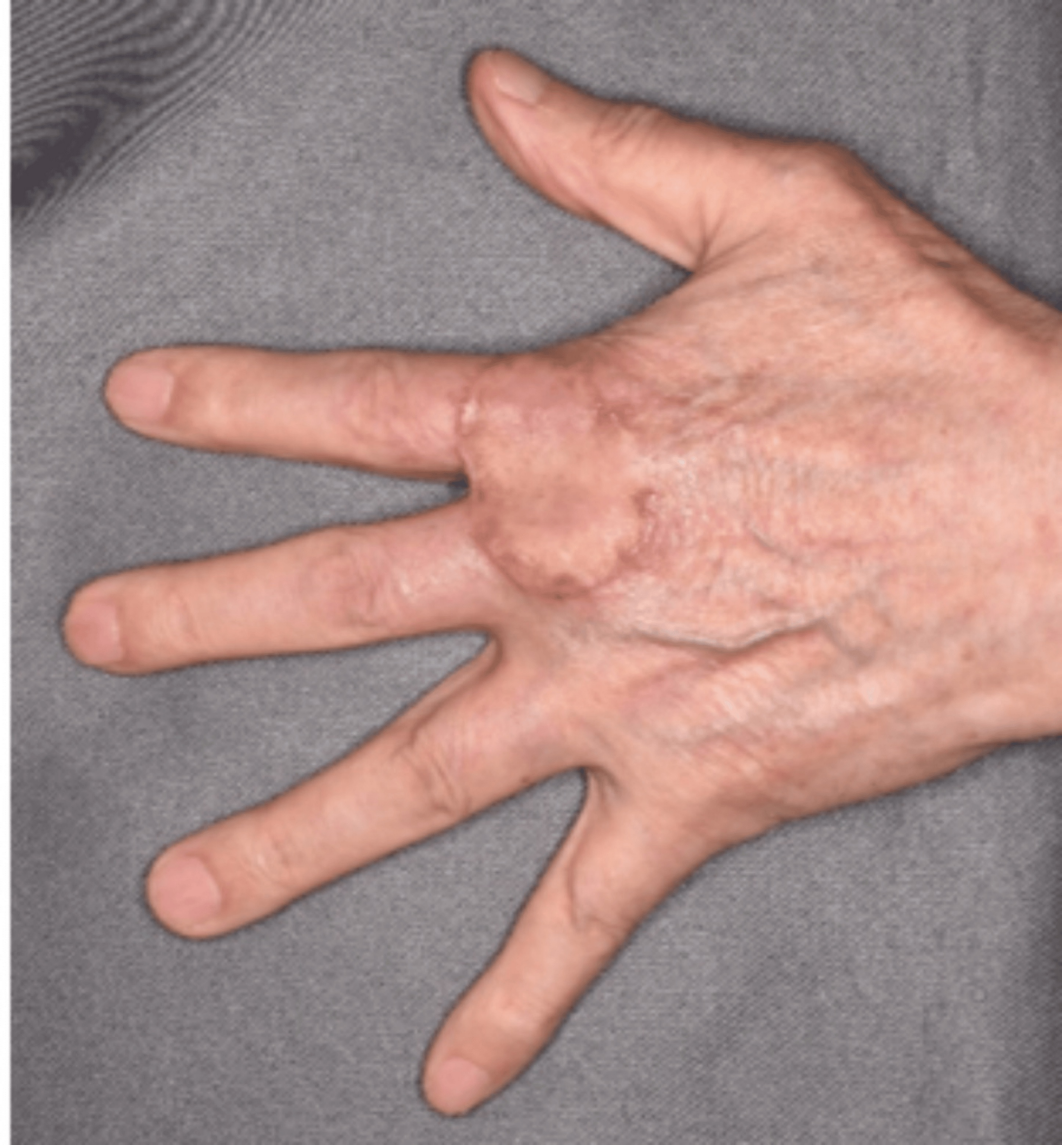
Postoperative skin graft after five months

The tumor measured 50*40 mm, and a surgical margin of 5 mm from the edge of the tumor was established for excision (Figures 1A, 1B). The depth of the excision extended just below the adipose layer (Figures 1C, 1D). A skin graft harvested from the right inguinal region was placed over the defect and securely fixed in position with tie-over sutures (Figure 1E). The pathological findings revealed Bowen’s disease with Merkel cell carcinoma; however, considering the negative margins and his older age, a watch-and-wait approach was adopted. Postoperatively, the patient is currently under outpatient observation, with no recurrence observed thus far. There were no significant issues with postoperative hand motor function and the skin graft has adhered successfully without any issues (Figure 2).

**Figure 3:**
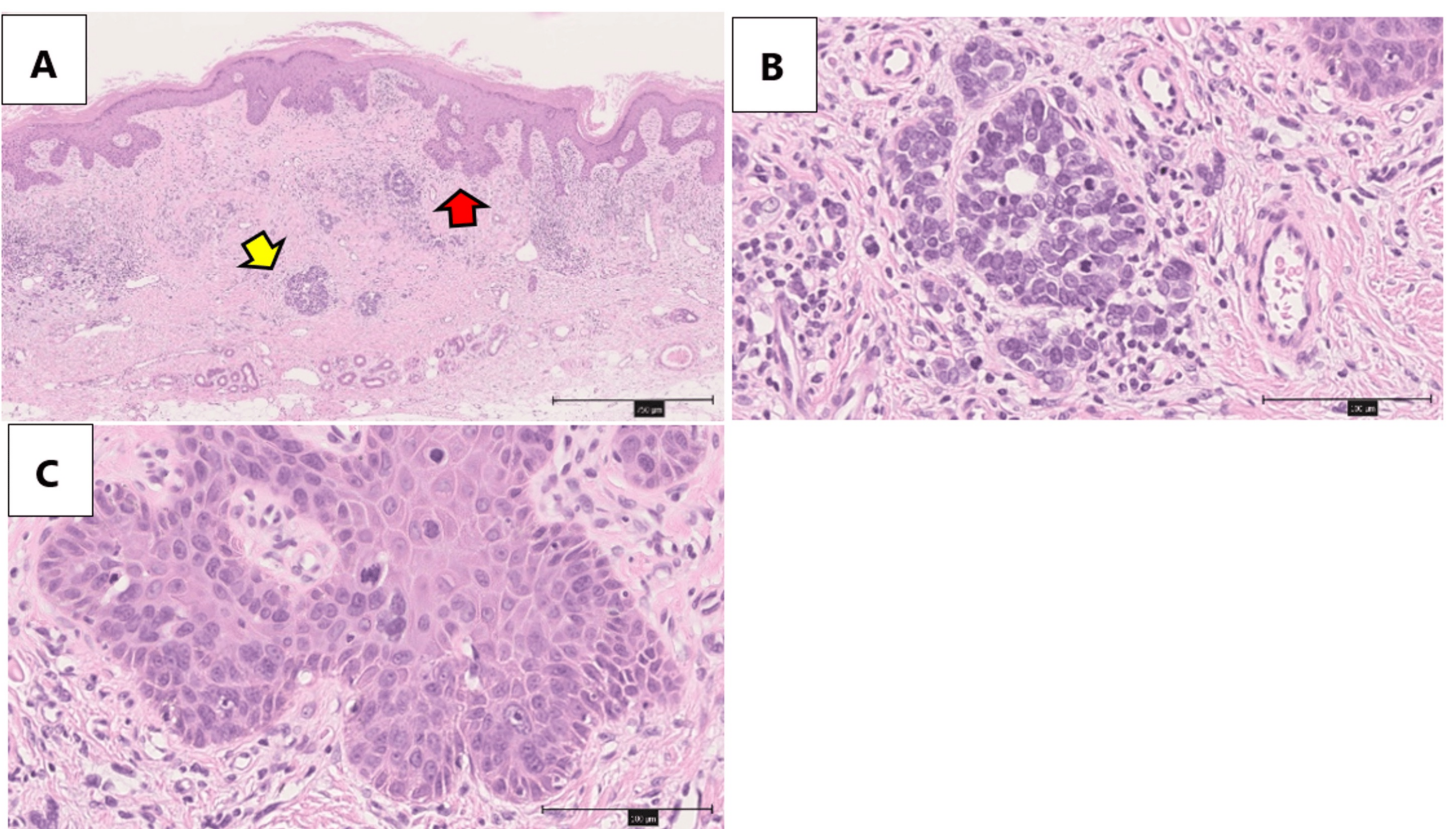
Hematoxylin and eosin (H&E) stain A: The yellow arrow shows the cytoplasm composed of small nests, which demonstrates Merkel cells. The red arrow shows Bowen's disease; B: Merkel cell; C: The nuclei showed pleomorphism and multinuclei cell, characteristic of Bowen's disease. Scale bars are 750 μm in A and 100 μm in B and C.

Hematoxylin and Eosin (HE) stains revealed that cells with varying sizes and shapes of nuclei proliferated prominently within the epidermis. Individual cell keratinization and atypical mitotic figures were also observed. In the dermis, areas were noted where cells with a high nuclear-to-cytoplasmic (N/C) ratio and inconspicuous cytoplasm formed small nests (Figures 3A, 3B).

**Figure 4:**
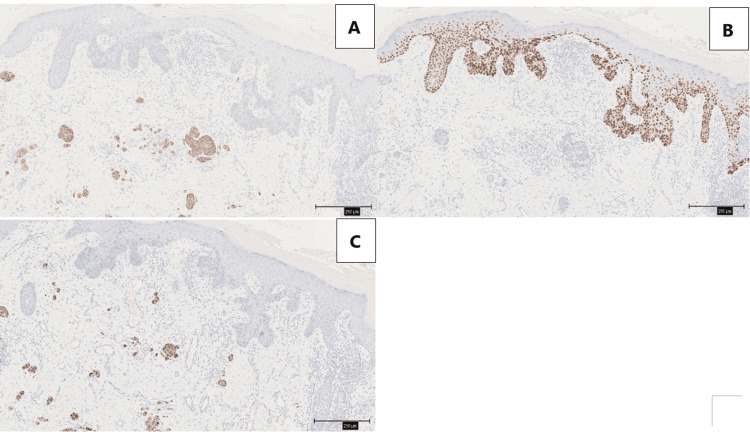
Immunohistochemistry A: Synaptophysin positive; B: p40 negative for MCC, positive for BD; C: positive for cytokeratin 20 (CK20), which shows a characteristic dot-like perinuclear staining pattern. Scale bars are all 250 μm. MCC: Merkel cell carcinoma

In immunohistochemistry, the subepidermal region showed positive staining for synaptophysin (Figure 4A), and negativity for p40 (Figure 4B). Cytokeratin 20 (CK20), which is specific to MCC, is distributed in a perinuclear dot cytoplasmic pattern (Figure 4C).

**Figure 5:**
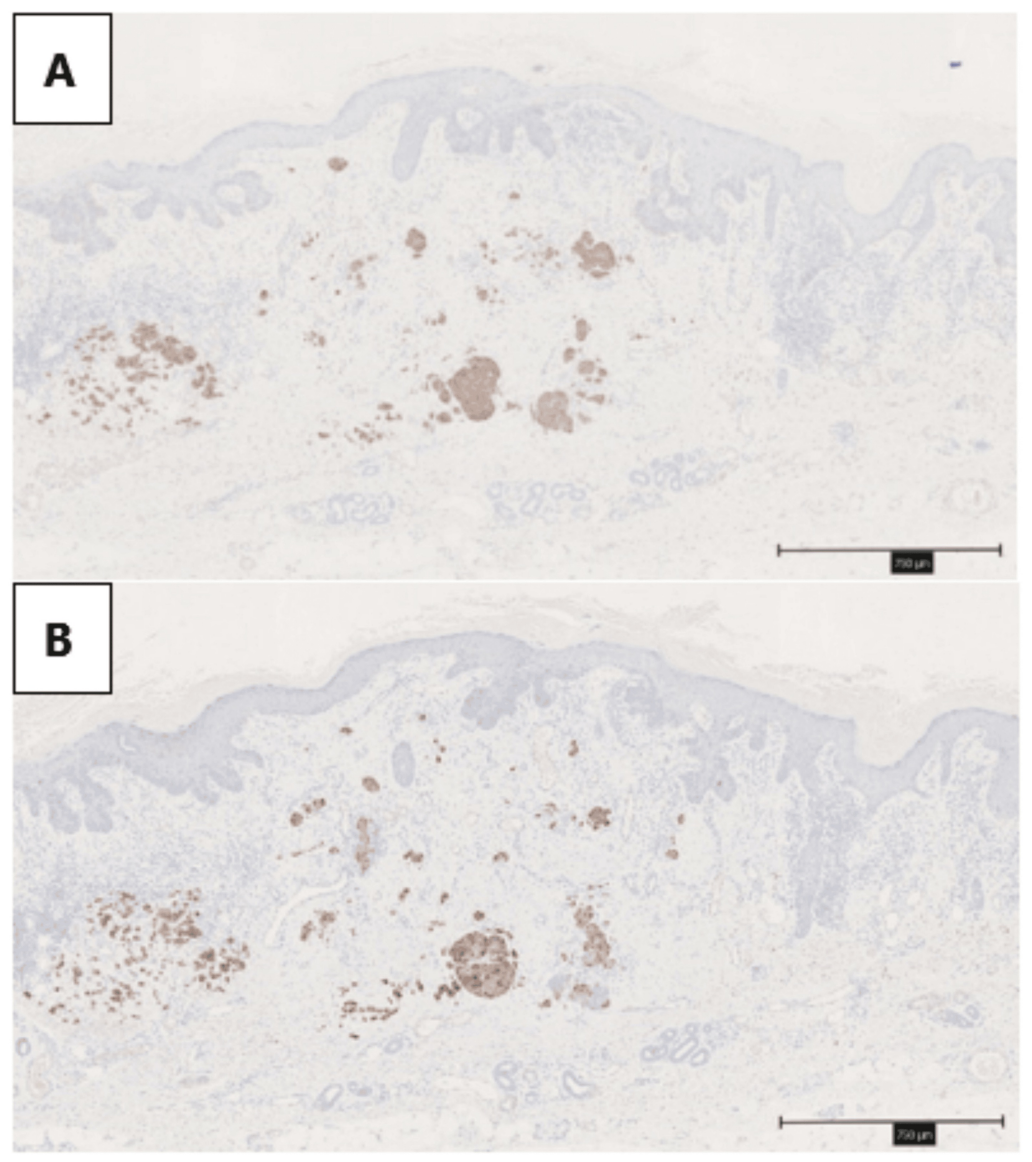
MCC was scattered within the dermis. A: Synaptophysin; B: CK20 Scale bars are all 750 μm. MCC: Merkel cell carcinoma

The MCC was scattered in small nests within the dermis (Figures 5A, 5B). In the findings of BD with MCC, there were no clear positive margins observed.

## Discussion

Cases where MCC coexists with BD are rare, and there are few case reports. Particularly, there are very few case reports that mention the primary site of MCC or involve locations other than the epidermis. A search on PubMed using the keywords 'Bowen’s disease' and 'Merkel cell carcinoma' or 'Squamous cell carcinoma' was conducted, focusing solely on case reports published from January 2015 to April 2024. This search yielded 12 relevant articles. Within these, 14 cases were identified (Table 1). Of these 14 cases, there were only 2 cases where the primary site of MCC was confined to the dermis or spanned from the epidermis to the dermis. Seven cases occurred in the head and neck region. It is widely recognized that more than half of MCC cases tend to occur in the head and neck region, followed by the extremities and trunk. Furthermore, there is a higher incidence among females compared to males [2,3].

**Table 1:** A literature review was conducted on case reports documenting the coexistence of BD and MCC from January 2015 to April 2024

Reference	year	Age/Sex	Site	Metastases	Diagnosis	Primary site
Chen X et al. [3]	2024	51/M	Right waist	No	MCC/BD	Epidermis
		87/F	Right mandible	No	MCC/BD	Epidermis
Jeha GM et al. [4]	2024	83/M	Neck	Yes	MCC/SCC in situ	Subcutis
Swain et al. [5]	2022	32/F	Right dorsum of the hand	Yes	MCC/BD	Epidermis
Kiyohara T et al. [6]	2019	65/M	Left leg	Yes	MCC/BD	Dermis to the subcutis
Yang A et al. [7]	2019	71/M	Right cheek	unreported	MCC/SCC in situ	Epidermis
Casari A et al. [8]	2018	85/F	Left cheek	unreported	MCC/BD	Epidermis
Czapiewski P et al. [9]	2016	76/F	cheek	Yes	MCC/SCC in situ	No described
		77/M	capillitium	No	MCC/BD	No described
Miraflor AP et al. [10]	2016	71/M	Left zygoma	No	MCC/BD	Epidermis
McGowan et al. [11]	2016	73/F	Right mandible	No	MCC/SCC in situ	Epidermis
Chou et al. [12]	2016	77/F	Right breast	Yes	MCC/BD	Epidermis
Tono et al. [13]	2015	71/M	back	unreported	MCC/BD	No described
Schick et al. [14]	2015	93/F	Left mandible	No	MCC/SCC in situ	Dermis

In the only systematic review conducted in Japan on MCC, coexistence with malignant tumors was observed in 87 out of 847 cases, with BD present only in 51 cases over 30 years in Japan. There were very few cases of MCC reported where subcutaneous nodules appear as the initial presentation without surface changes. Interestingly, their findings suggest that subcutaneous MCC in Japanese patients manifests as a localized disease with a positive prognosis and could be a prognostic factor associated with improved overall survival [2]. Previous reports of BD combined with MCC consistently show large nodules of atypical cells. However, this case presents numerous small micro-nodules in the dermis, which is extremely rare in this context. The pathogenesis of MCC and its association with BD remain unclear, but the absence of large nodules in this case suggests that a very early-stage MCC developed beneath BD and was incidentally detected by biopsy.

Recently, the association between Merkel cell polyomavirus (MCPyV) and MCC has attracted significant scholarly interest. MCPyV-positive MCC is associated with a better prognosis [15]. Furthermore, there are reports that most MCCs with characteristics of epithelial tumors, such as squamous cell carcinoma, referred to as combined MCC, are MCPyV-negative [16]. Our institution lacks the testing devices for MCPyV, and thus we have not examined the relationship between the current patient and MCPyV, which represents a limitation. However, given that the lesion is on the dorsum of the hand, an area exposed to sunlight over 30 years due to his job, and is concurrent with BD, which has characteristics of epithelial tumors, it is presumed to be more likely caused by sun exposure rather than MCPyV positivity. If this hypothesis is considered, the present case is highly likely to be MCPyV-negative, indicating an unfavorable prognosis. In terms of prognosis and additional treatment, clinical testing for MCPyV may become essential in the future.

Regarding the surgical procedure, this case was diagnosed as BD via excisional biopsy; therefore, surgical excision with a 5 mm margin was performed. When Merkel cell carcinoma is suspected, a margin of 1-2 cm is recommended [17]. In this case, BD was initially suspected, leading to excision with a 5 mm margin. However, if MCC had been suspected initially, a margin of 1-2 cm would have been considered. Additional excision and radiation therapy were considered, but no further treatment was performed because he was an elderly male patient, the margins were negative at the initial surgery, and no lymph node metastasis was observed. Given the high local recurrence rate of MCC [7], ongoing outpatient follow-up is necessary. Six months have passed since the surgery, and no recurrence has been observed at this time.

## Conclusions

This case report presents a particularly rare instance of BD coexisting with intradermal MCC located primarily in the dermis of the dorsum of the hand in an elderly Japanese male. The unusual presentation of MCC in the subcutaneous layer, rather than the more common epidermal locations, makes this case exceptionally uncommon and noteworthy.

This report not only highlights the unique subcutaneous presentation but also emphasizes the need for careful diagnostic evaluation when encountering complex or unusual skin lesions. The findings add valuable knowledge to the limited literature on subcutaneous MCC and its coexistence with Bowen’s disease, stressing the importance of vigilance in such rare and challenging cases. Further research may be warranted to explore the underlying mechanisms and optimal management strategies for similar cases.
